# Protection of Mice Orally Infected With Shiga Toxin–Producing *Citrobacter rodentium* Treated With Nanobody Multimer

**DOI:** 10.1093/ofid/ofag357

**Published:** 2026-06-06

**Authors:** Abhineet Sheoran, Amanda J Martinot, Sally R Robinson, Anne Fu, Cara Martone, Denise Dayao, Charles B Shoemaker, Saul Tzipori

**Affiliations:** Department of Infectious Disease and Global Health, Cummings School of Veterinary Medicine, Tufts University, North Grafton, Massachusetts, USA; Department of Infectious Disease and Global Health, Cummings School of Veterinary Medicine, Tufts University, North Grafton, Massachusetts, USA; Department of Infectious Disease and Global Health, Cummings School of Veterinary Medicine, Tufts University, North Grafton, Massachusetts, USA; Department of Infectious Disease and Global Health, Cummings School of Veterinary Medicine, Tufts University, North Grafton, Massachusetts, USA; Department of Infectious Disease and Global Health, Cummings School of Veterinary Medicine, Tufts University, North Grafton, Massachusetts, USA; Department of Infectious Disease and Global Health, Cummings School of Veterinary Medicine, Tufts University, North Grafton, Massachusetts, USA; Department of Infectious Disease and Global Health, Cummings School of Veterinary Medicine, Tufts University, North Grafton, Massachusetts, USA; Department of Infectious Disease and Global Health, Cummings School of Veterinary Medicine, Tufts University, North Grafton, Massachusetts, USA

**Keywords:** camelid nanobody, *Citrobacter rodentium*, hemolytic-uremic syndrome, Shiga toxin–producing *Escherichia coli*; STEC

## Abstract

**Background:**

Shiga toxin (Stx)–producing *Escherichia coli* (STEC) colonizes the gut and causes enteritis through bacterial attachment-effacement (A/E), leading to mucosal damage. This facilitates systemic uptake of Stx, which can often lead to hemolytic-uremic syndrome (HUS) and acute renal failure, particularly in children <5 years of age. STEC infections expressing Stx2 variants are a major risk factor for HUS. Beyond patients receiving supportive care, there is no treatment for STEC-mediated HUS, and antibiotics are contraindicated. The 3- to 7-day prodromal interval between onset of STEC diarrhea and HUS offers a window for intervention for diarrheic patients, contacts thereof, or individuals exposed to the source of infection.

**Methods:**

*Citrobacter rodentium* (*Cr*), a mouse pathogen that exhibits mucosal bacterial A/E, was genetically constructed to express Stx2d (*Cr*-Stx2d). This, along with a *Cr* parent strain carrying the kanamycin gene, were used to model STEC in mice. Mice were treated intraperitoneally with a broad subtype-specific, Stx-neutralizing nanobody multimer fused to the human IgG1 Fc domain (VNA2-Stx/hFc).

**Results:**

When a single treatment was administered as late as 4 days after *Cr*-Stx2d challenge, likely within the human STEC-HUS window of intervention, complete protection of mice was achieved.

**Conclusions:**

This *Cr*-Stx2d model, which mimics elements of human STEC infections by causing mucosal gut lesions and systemic Stx2d-mediated kidney damage, will help evaluate specific treatments against STEC-HUS, as demonstrated here with VNA2-Stx/hFc-treated mice given a single injection well after bacterial challenge. This product should be safe, simple, and economical to manufacture.

## Teaser

Shiga toxin–producing *Escherichia coli* causes bloody diarrhea and can lead to hemolytic-uremic syndrome. We developed an antitoxin nanobody that protects mice when given systemically ≥4 days after challenge with mouse enteric *Citrobacter rodentium* expressing Shiga toxin.

Infection of humans with Shiga toxin (Stx)–producing *Escherichia coli* (STEC), the source of which is mostly bacterial contamination of food products, can cause severe enteric and systemic disease manifested by bloody diarrhea and mucosal damage characterized by attaching-effacing (A/E) lesions. A serious sequelae of STEC, hemolytic-uremic syndrome (HUS) is a leading cause of childhood acute renal failure [1–4]. Epidemiological studies show that STEC expressing Stx2 variants Stx2a, Stx2c, and Stx2d are responsible for HUS [5]. No specific treatment is available for HUS, and antibiotics are contraindicated [6]. Systemic administration of Stx-specific neutralizing agents are the most promising approaches for prevention or treatment of STEC-HUS.

Animal models used to demonstrate the efficacy of Stx-specific neutralizing antibodies include (1) a mouse toxicity model where Stx is directly administered systemically [7–9], causing rapid mortality; (2) mice that are pretreated with streptomycin and orally infected with STEC [9, 10]; and (3) gnotobiotic piglets orally challenged with STEC [6, 11–13]. STEC in piglets induces severe A/E lesions but no kidney lesions are observed. Gnotobiotic piglets lack gut microbiota and are agammaglobulinemic, making them extremely susceptible to STEC infections and to other pathogens [6, 11–16].

Given the limitations of current STEC animal models, here we optimize the Stx–*Citrobacter rodentium* (*Cr*) mouse model [17–20] to evaluate Stx-specific immunotherapy. *Cr*, a natural pathogen of mice that exhibits A/E mucosal lesions, was genetically modified to express Stx2dact (hereafter “Stx2d”) and, as a result, elicits a lethal disease of the gut that includes kidney damage [17, 18]. As in humans, mice orally infected with Stx2d-expressing *Cr* (*Cr*-Stx2d) produce mucosal A/E lesions and damage kidney tubules. In contrast, the lesions in humans are glomerular [17, 18].

We demonstrate the utility of this *Cr*-Stx2d mouse model to test the efficacy of our Stx-neutralizing nanobody heterotetramer construct (Variable Heavy domain of a Heavy-chain only antibody [VHH]-based neutralizing agent [VNA]) in a clinically relevant, postinfection treatment model. This VNA has previously been shown to effectively neutralize 3 Stx types (Stx1, Stx2a, and Stx2d) in cell culture and in mouse toxicity assays [20, 21]. With the *Cr*-Stx2d mouse model, we demonstrate full protection against fatal disease after a single parenteral VNA treatment, even when treated several days after bacterial challenge.

## MATERIALS AND METHODS

### Ethics Statement

In vivo studies were performed in accordance with the Guide for the Care and Use of Laboratory Animals of the National Institutes of Health and were approved by the Institutional Animal Care and Use Committees (Protocol G2024-100), with Animal Welfare Assurance Number D16-00572 (A4059-01).

### VNA2-Stx/hFc VNA

It consists of a VHH heterotetramer fused with the human IgG1 Fc to prolong serum half-life of the VNA. In combination, the 4 VHH components neutralize Stx1, Stx2a, and Stx2d [6, 21]. VNA2-Stx/hFc was produced and purified by Genscript in Chinese hamster ovary cells using the vector pcDNA3.4, with the leader sequence MGWSCIILFLVATATGVHS. Purified VNA2-Stx/hFc (2.51 mg/mL) in phosphate-buffered saline (PBS) was stored at −20°C. Sodium dodecyl sulphate–polyacrylamide gel electrophoresis demonstrated that the protein was 95% pure with a molecular weight of approximately 78 kDa in reducing conditions and approximately 170 kDa in nonreducing conditions.

### Mouse Model of Stx2d-Producing *C rodentium* for Determining Efficacy of VNA2-Stx/hFc Treatment

*Citrobacter rodentium–*expressing Stx2d (*Cr-*Stx2d) and the *Cr* parent strain carrying the kanamycin gene instead of the Stx2d gene were used to infect mice as described elsewhere [19].

Five-week-old male and female C57BL/6 mice (Jackson Laboratory) were divided into groups of 3–4 per group (or 8 per group when indicated) and fasted for approximately 12 hours before oral infection. Sterile rodent chow was broken up into pieces of approximately 40 mg, and 6 µL of bacterial suspension containing approximately 1 × 10^8^  *Cr* or *Cr*-Stx2d was dispensed onto each piece. Mice were individually presented with inoculated chows in respective containers and returned to their standard cages after they were observed to have consumed the entire piece.

Groups of mice inoculated with *Cr*-Stx2d then later received a single treatment of 25 µg/mouse VNA2-Stx/hFc intraperitoneally (IP) at different days postinfection, from day 0 through day 6. Control groups of infected mice received human IgG1 (25 µg/mouse) or PBS. Male or female mice that received neither the infection nor the treatment were used as additional controls. Mice were monitored daily for clinical signs of lethargy, neurologic signs, and body weight changes. Mice displaying ataxia or >15% body weight loss were euthanized. Feces were collected on day 5 postinfection to establish bacterial amplification in the gut, and surviving mice were euthanized 19–21 days postinfection. After euthanasia, blood and tissue were collected to determine serum VNA2-Stx/hFc, blood urea nitrogen (BUN), and creatinine levels. The colon and kidney were examined histologically for evidence of mucosal and kidney lesions.

### Enzyme-Linked Immunosorbent Assays to Measure Serum VNA2-Stx/hFc Levels

This assay was performed as previously described [22] with the following modifications. Plates were coated with Stx2a and then incubated with a dilution series of test serum. After washing, the Stx2a-bound VNA2-Stx/hFc was detected with 1:4000 diluted goat anti-human IgG Fc horseradish peroxidase (Southern Biotech). A dilution series of VNA2-Stx/hFc was employed to produce a protein standard curve and Gen5 software was used to quantify serum VNA2-Stx/hFc levels.

### Statistical Analysis

Statistical analyses were performed using Prism version 10 software (GraphPad). For comparisons of bacterial burden in feces on day 5 postinfection, serum VNA2-Stx/hFc levels and body weight differences on the final day of the experiment were employed. A one-way analysis of variance with post hoc Tukey honest significant difference analysis was used to determine statistical significance between the groups. Survival curves were assessed using the Mantel–Cox log-rank test. Resulting *P* values of ≤.05 were considered significant.

## RESULTS

### Protective Efficacy of VNA2-Stx/hFc Treatment of Mice 2 Hours (Day 0) Post–*Cr*-Stx2d Oral Challenge

Mouse experimental groups are outlined in Table 1. On day 0 postchallenge, the treatment groups received an IP dose of 25 μg of purified VNA2-Stx/hFc, while the control groups received 25 μg of human IgG1. Fecal samples were collected on day 5 postinfection to confirm bacterial colonization. All surviving animals were humanely euthanized 19 days after challenge with *Cr* or *Cr*-Stx2d.

**Table 1.** Experimental Design for Determining Protective Efficacy of VNA2-Stx/hFc Administered Day 0 Postinfection

Group/Sex	Oral Challenge	Treatment	Mouse, No.	Mouse Survived, No.
1/F	None	None	3	3
1/M	None	None	3	3
2/F	*Cr*	Human IgG1	4	4
2/M	*Cr*	Human IgG1	4	4
3/F	*Cr*-Stx2d	Human IgG1	4	0
3/M	*Cr*-Stx2d	Human IgG1	4	0
4/F	*Cr*-Stx2d	VNA2-Stx/hFc	8	8
4/M	*Cr*-Stx2d	VNA2-Stx/hFc	8	8

Abbreviations: *Cr*, *Citrobacter rodentium*; F, female; hFc, human IgG1 Fc domain; M, male; Stx2d, Shiga toxin 2d; VNA2, Variable Heavy domain of a Heavy-chain only antibody neutralizing agent 2.

**Figure 1.**
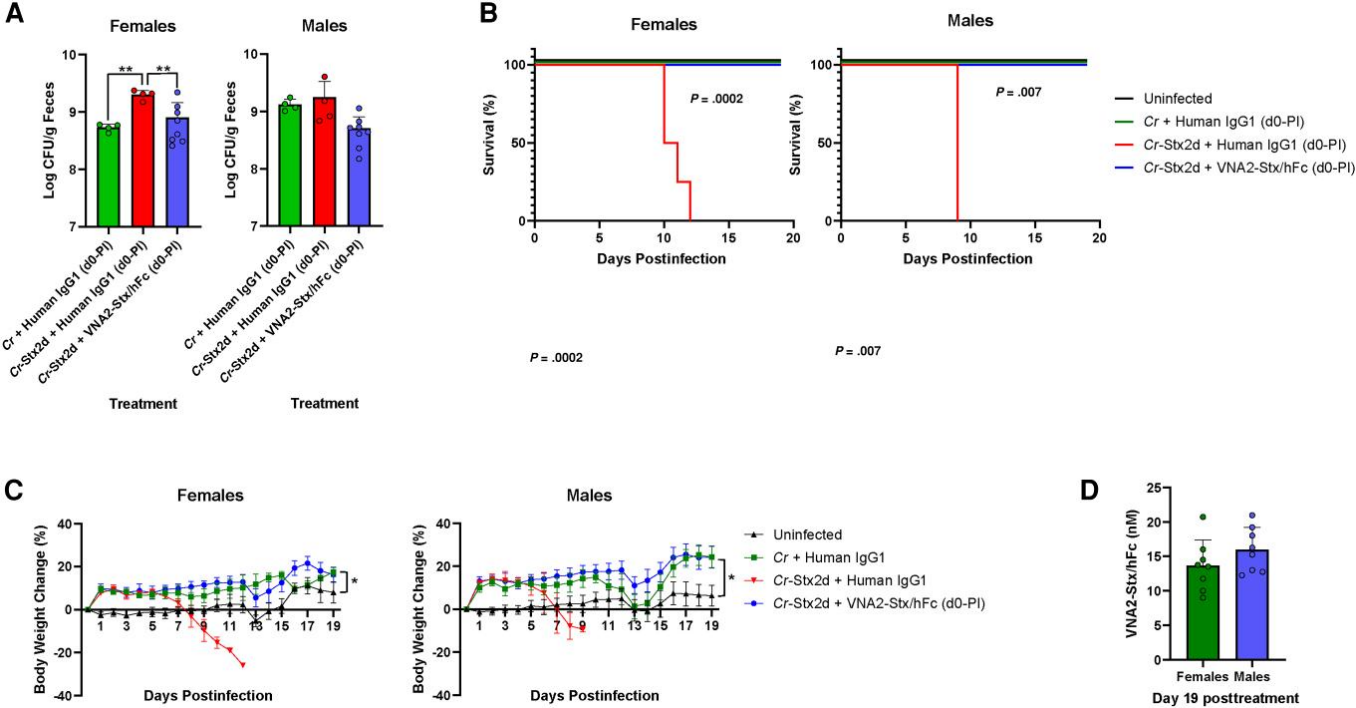
VNA2-Stx/hFc treatment day 0 post–oral infection with Stx2d-producing *Citrobacter rodentium* (*Cr*) protects all mice. Groups of male (M) and female (F) mice were infected orally with approximately 1 × 10^8^ CFU of either non-Stx-producing *Cr* (n = 4F, 4M) or Stx2d-producing *Cr* (*Cr*-Stx2d, n = 4F, 4M), and then received a single intraperitoneal 25 µg/mouse dose of either human IgG1 (n = 4F, 4M) or VNA2-Stx/Fc (n = 8F, 8M) within ∼2 h of the infection (called day 0 postinfection [d0-PI]). *A*, Colonization of the gut by *Cr* or *Cr*-Stx2d on day 5 PI is shown for female and male mice. Several fecal pellets were collected and assessed for total *Cr* bacteria numbers, ie, CFU/g of fecal material. The mean + SD (error bar) is also displayed. Fecal shedding of *Cr* by each mouse is represented by a circle. Statistically significant differences where observed are indicated as **P < .005. *B*, Kaplan–Meier survival plots of the different groups of mice. Mice were monitored daily and euthanized if they had ataxia, reluctance to move, or opisthotonos or lost >15% body weight. In contrast, all female and male mice that received VNA2-Stx/hFc treatment day 0 postchallenge survived (*P* values as shown). *C*, The percent change in body weights of mice of different groups are shown (mean ± SD). Body weights among surviving female or male mice between *Cr* + human IgG1 and *Cr*-Stx2d + VNA2-Stx/hFc groups at the end of the experiment did not differ significantly. Unexpectedly, mice in the uninfected control groups did not show body weight increases over the first week of the experiment, and therefore, the weights in uninfected female or male mice were significantly lower than those of the female or male mice in the *Cr* + human IgG1 and *Cr*-Stx2d + VNA2-Stx/hFc groups (*P < .01). *D*, Serum VNA2-Stx/Fc levels in nanomolar (mean + SD and individual values in circles) were assessed using dilution enzyme-linked immunosorbent assays on serum collected from all surviving mice at the end of the study (day 19 post–IP administration). For comparisons of the bacterial burden in feces (*A*), body weight differences on the final day of the experiment (*C*), and serum VNA2-Stx/hFc levels (*D*), one-way ANOVA with post hoc Tukey honest significant difference analysis was used to determine statistical significance between the groups. Survival curves were assessed using the Mantel–Cox log-rank test. Abbreviations: CFU, colony-forming units; *Cr*, *Citrobacter rodentium*; hFc, human IgG1 Fc domain; PI, postinfection; SD, standard deviation; Stx, Shiga toxin; Stx2d, Shiga toxin 2d; VNA2, Variable Heavy domain of a Heavy-chain only antibody neutralizing agent 2.

Bacterial gut amplification was extensive in all infected mice on day 5 (Figure 1*A*). There was no significant difference in the extent of bacterial excretion in the feces among male mice groups. Within the female mice groups, however, the bacterial burden in the *Cr* + human IgG1 group was significantly lower than the *Cr*-Stx2d + human IgG1 control treatment group (*P* = .004) (Figure 1*A*). The difference between these 2 groups could be due to Stx2d enhancing mucosal colonization. The control female *Cr*-Stx2d + human IgG1 group had significantly higher bacterial burden than the VNA2-Stx/hFc-treated females (*Cr*-Stx2d + VNA2-Stx/hFc group) (*P* = .006). However, in both groups, there were several mice that excreted bacteria in the feces to a similar extent, but they had different outcomes. VNA2-Stx/hFc treatment protected all mice while human IgG1 control protected none (Figure 1*B*). The results show that the protective efficacy of VNA2-Stx/hFc is not associated with a lower bacterial excretion in the *Cr*- Stx2d + VNA2-Stx/hFc group. All mice that received human IgG1 control treatment and *Cr*-Stx2d challenge died or were euthanized due to severe clinical signs; females by day 12 postinfection, males by day 9 (Figure 1*B*). In contrast, all female (*P* = .0002) and male (*P* = .007) mice that received VNA2-Stx/hFc treatment day 0 postchallenge survived. In addition, all mice that received non-Stx-producing *Cr* survived, indicating that bacterial amplification of *Cr* lacking Stx2d expression did not induce severe illness or mortality. Although this group of mice all survived, there was some weight loss, presumably due to pathology from A/E mucosal lesions.

The body weights of mice that did not survive began to decrease after 4–5 days of *Cr*-Stx2d challenge and rapidly declined thereafter (Figure 1*C*), whereas the body weights of mice that survived *Cr*-Stx2d challenge, due to VNA2-Stx/hFc treatment, generally increased. All mice in the control groups that received non-Stx-producing *Cr*, as well as the group receiving *Cr*-Stx2d, survived and displayed similar weight profiles. However, mice in all groups except one (*Cr*-human IgG1 females) showed a similar, unexplained drop in body weight on day 13 postinfection. There was no significant difference in weights among female or male mice between the *Cr* + human IgG1 and *Cr*-Stx2d + VNA2-Stx/hFc groups at the end of the experiment. Unexpectedly, mice in the uninfected control groups did not show body weight increases over the first week of the experiment, and therefore, the weights in uninfected female or male mice were significantly lower than those of the female or male mice in the *Cr* + human IgG1 and *Cr*-Stx2d + VNA2-Stx/hFc groups (*P*  *≤* .007). This lack of weight increase may be related to stress from the substantial handling required for these studies. Control groups receiving *Cr* alone were initially included to test the effect of A/E on the gut mucosa in the absence of Stx production, to be observed histologically at euthanasia. Since they all survived the *Cr* challenge, these groups were not included in the subsequent experiments described below. All VNA2-Stx/hFc-treated female and male mice retained serum VNA antitoxin levels of approximately 10–20 nM when euthanized at 19 days post–IP administration (Figure 1*D*). The results demonstrate that VNA2-Stx/hFc treatment approximately 2 hours (day 0) after oral infection with *Cr*-Stx2d protected mice against fatal systemic intoxication.

Sixteen residual blood samples were submitted for BUN and creatinine analysis. Fourteen of them were taken at euthanasia from treated mice that were asymptomatic, and these serum markers measured well within the normal range of 17–27 mg/dL for BUN and 0.1 mg/dL for creatinine (range, 0.1–1.0), indicating robust protection against death and long-term tissue damage. Unfortunately, all but 2 of the symptomatic control animals died overnight and could not be tested. One of the 2 was azotemic, with elevated BUN at 156 mg/dL and creatinine of 0.6 mg/dL, while the second one had levels just above normal range for 28 mg/dL for BUN and within normal at 0.1 mg/dL for creatinine.

### Protective Efficacy of VNA2-Stx/hFc Treatment on Day 1, 2, or 3 Post–*Cr*-Stx2d Oral Challenge in Mice

**Figure 2.**
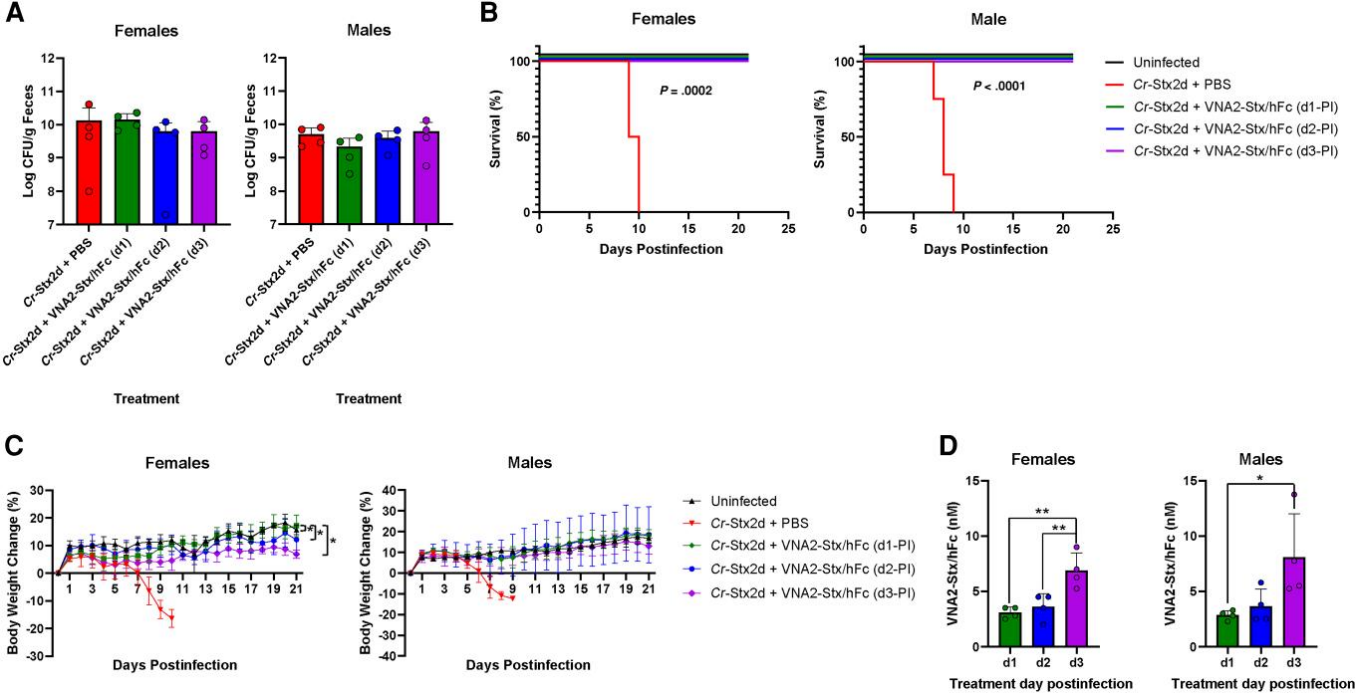
VNA2-Stx/hFc treatment days 1–3 post–oral infection with Stx2d-producing *Citrobacter rodentium* (*Cr*) protects all mice. Groups of male and female mice were infected orally with approximately 1 × 10^8^ CFU of Stx2d-producing *Cr* (*Cr*-Stx2d) and received PBS on day 1 postinfection or a single 25 µg/mouse of VNA2-Stx/hFc IP on day 1, 2, or 3 after the infection. Each male or female mouse group had 4 mice except for uninfected male or female groups, which had 3 mice each. *A*, Colonization of the gut by *Cr*-Stx2d in female and male mice displayed no statistically significant differences among the 4 groups. Results are expressed as mean + SD (error bar) CFU/g feces measured on day 5 postinfection. Fecal shedding of the organisms by each mouse is also shown (circles). *B*, Kaplan–Meier survival plots of the different groups of mice. Mice were monitored daily and euthanized if they had ataxia, reluctance to move, or opisthotonos or lost >15% body weight. All female and male mice that received VNA2-Stx/hFc treatment days 1–3 postchallenge survived (*P* values as shown). *C*, The percent change in body weights of mice of different groups are shown (mean ± SD). At the end of the experiment, no significant differences were observed in body weights of any of the surviving male and female mice groups except between uninfected female and treated female groups (*P  *<* .05). *D*, Serum VNA2-Stx/hFc levels in nanomolar (mean + SD and individual values in circles) and statistically significant differences (*P  *<* .05; **P  *<* .01) are shown at the end of the experiment (day 19 post–IP administration). For comparisons of the bacterial burden in feces (*A*), body weight differences on the final day of the experiment (*C*), and serum VNA2-Stx/hFc levels (*D*), one-way ANOVA with post hoc Tukey honest significant difference analysis was used to determine statistical significance between the groups. Survival curves were assessed using the Mantel–Cox log-rank test. Abbreviations: CFU, colony-forming units; *Cr*, *Citrobacter rodentium*; hFc, human IgG1 Fc domain; IP, intraperitoneally; PBS, phosphate-buffered saline; PI, postinfection; SD, standard deviation; Stx, Shiga toxin; Stx2d, Shiga toxin 2d; VNA2, Variable Heavy domain of a Heavy-chain only antibody neutralizing agent 2.

Mouse experimental groups are outlined in Table 2. All surviving animals were humanely euthanized 21 days after challenge with *Cr*-Stx2d. *Cr*-Stx2d proliferation was observed in the feces (Figure 2*A*) and displayed no significant differences among the 4 groups. Control groups receiving the human IgG1 treatment and challenged with *Cr*-Stx2d either died or were euthanized due to severe clinical signs by days 10 and 9 postinfection, respectively (Figure 2*B*). In contrast, mice receiving the VNA2-Stx/hFc treatment on days 1, 2, or 3 postchallenge survived (*P*  *≤* .0002). The body weights of mice that did not survive continued to decrease over the following 3–5 days after *Cr*-Stx2d challenge (Figure 2*C*). Body weights of mice that survived *Cr*-Stx2d challenge due to VNA2-Stx/hFc treatment increased and were higher than their starting weight at the end of the experiment. As expected, mice in the uninfected control groups also increased their body weights. There was no significant difference in body weights of any of the male mice groups at the end of the experiment. The only significant differences in the final weights for the female mice groups were between the uninfected and *Cr*-Stx2d + VNA2-Stx/hFc (day 3 postinfection) groups (*P*  *=* .045), and between the *Cr*-Stx2d + VNA2-Stx/hFc (day 1 postinfection) and *Cr*-Stx2d + VNA2-Stx/hFc (day 3 postinfection) groups (*P*  *=* .011). All treated mice still had VNA2-Stx/hFc serum concentration of 2–10 nM when euthanized 18–20 days post–IP administration (Figure 2*D*). These results demonstrate that VNA2-Stx/hFc treatment, when administered IP 1–3 days postinfection with *Cr*-Stx2d, still fully protected mice against fatal systemic intoxication.

**Table 2.** Experimental Design for Protective Efficacy Studies With VNA2-Stx/hFc Administered Days 1–3 Post–*Cr*-Stx2d Challenge

Group/Sex	Oral Challenge	Treatment	Treatment Day Postinfection	Mouse, No.	Mouse Survived, No.
1/F	None	None	None	3	3
1/M	None	None	None	3	3
2/F	*Cr*-Stx2d	PBS	0	4	0
2/M	*Cr*-Stx2d	PBS	0	4	0
3/F	*Cr*-Stx2d	VNA2-Stx/hFc	1	4	4
3/M	*Cr*-Stx2d	VNA2-Stx/hFc	1	4	4
4/F	*Cr*-Stx2d	VNA2-Stx/hFc	2	4	4
4/M	*Cr*-Stx2d	VNA2-Stx/hFc	2	4	4
5/F	*Cr*-Stx2d	VNA2-Stx/hFc	3	4	4
5/M	*Cr*-Stx2d	VNA2-Stx/hFc	3	4	4

Abbreviations: *Cr*, *Citrobacter rodentium*; F, female; hFc, human IgG1 Fc domain; M, male; PBS, phosphate-buffered saline; Stx2d, Shiga toxin 2d; VNA2, Variable Heavy domain of a Heavy-chain only antibody neutralizing agent 2.

### Protective Efficacy of VNA2-Stx/hFc Treatment Given on Day 4, 5, or 6 Post–*Cr*-Stx2d Oral Challenge in Mice

**Figure 3.**
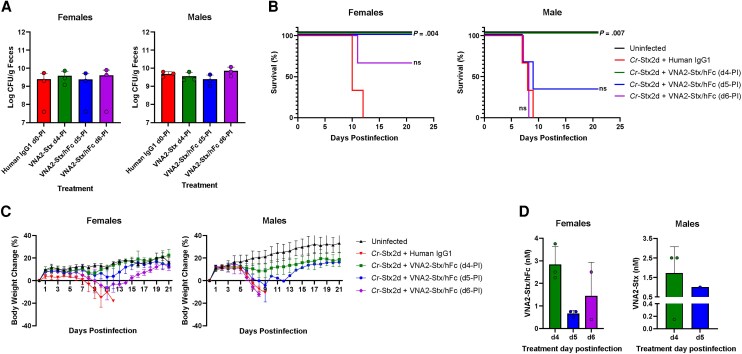
VNA2-Stx/hFc treatment days 4–6 post–oral infection with Stx2d-producing *Citrobacter rodentium* (*Cr*) protects mice up to ≥4 days postinfection. Groups of male and female mice were infected orally with approximately 1 × 10^8^ CFU of Stx2d-producing *Cr* (*Cr*-Stx2d) and received a single dose of 25 µg/mouse of human IgG1 on day 1 postinfection or of VNA2-Stx/hFc intraperitoneally on day 4, 5, or 6 after the infection. Each male or female mouse group had 3 mice. *A*, Colonization of the gut by *Cr*-Stx2d in female and male mice displayed no statistically significant differences among the 4 groups. Results are expressed as mean + SD (error bar) CFU/g feces measured on day 5 postinfection. Fecal shedding of the organisms by each mouse is also shown (circles). *B*, Kaplan–Meier survival plots of the different groups of mice. Mice were monitored daily and euthanized if they had ataxia, reluctance to move, or opisthotonos or lost >15% body weight. Female mice treated with VNA on days 4 and 5 but not on day 6 postinfection were completely protected (*P* values as shown; “ns” indicates nonsignificant). In contrast, males treated with VNA on day 4 but not on days 5 and 6 postchallenge were completely protected (*P* values as shown; “ns” indicates nonsignificant). *C*, The percent change in body weights of mice of different groups are shown (mean ± SD). There was no statistically significant difference between any of the surviving groups at the end of the experiment. *D*, Serum VNA2-Stx/hFc levels in nanomolar (mean + SD and individual values in circles) are shown at the end of the experiment (day 19 post–IP administration). There was no statistically significant difference between any groups. For comparisons of the bacterial burden in feces (*A*), body weight differences on the final day of the experiment (*C*), and serum VNA2-Stx/hFc levels (*D*), one-way ANOVA with post hoc Tukey honest significant difference analysis was used to determine statistical significance between the groups. Survival curves were assessed using the Mantel–Cox log-rank test. Abbreviations: CFU, colony-forming units; *Cr*, *Citrobacter rodentium*; hFc, human IgG1 Fc domain; IP, intraperitoneally; PI, postinfection; SD, standard deviation; Stx, Shiga toxin; Stx2d, Shiga toxin 2d; VNA2, Variable Heavy domain of a Heavy-chain only antibody neutralizing agent 2.

Three mice per group received VNA2-Stx/hFc treatment (25 μg dose IP per mouse), on day 4, 5, or 6 post–*Cr*-Stx2d oral challenge. All surviving animals were humanely euthanized 21 days after challenge. *Cr*-Stx2d amplification was evident in all mouse feces (Figure 3*A*), showing no significant differences among the groups. Mice that were treated with control human IgG1 and challenged with *Cr*-Stx2d all died or were euthanized due to severe clinical disease by day 12 postinfection in the female group and by day 9 in the male group (Figure 3*B*). All female mice treated with VNA2-Stx/hFc on days 4 and 5 postchallenge survived (*P*  *=* .004), while 2 of 3 female mice survived when treated on day 6 (*P*  *=* .11). All males treated with VNA2-Stx/hFc on day 4 postchallenge survived (*P*  *=* .007), but only 1 of the 3 mice treated on day 5 survived. None of the mice treated on day 6 postchallenge (*P*  *=* .39) survived. The body weights of the infected control mice continued to drop during the 5 days of *Cr*-Stx2d challenge (Figure 3*C*), while the body weights of the surviving mice treated with VNA2-Stx/hFc increased to levels higher than their starting weight. In contrast, body weights of VNA2-Stx/hFc-treated mice that did not survive *Cr*-Stx2d challenge continued to decrease after 5–6 days of *Cr*-Stx2d challenge. As expected, the body weights of the mice in the uninfected control groups increased. There was no significant difference in body weights of any of the surviving male mice at the end of the experiment. All female and male mice showed easily detected serum levels of VNA2-Stx/hFc, although they were lower than those reported in prior studies, ranging from 0.1 to 4 nM on day 18–20 post–IP administration (Figure 3*D*). The small discrepancies in final VNA serum levels may be due to factors such as differences in time of sampling, sources, and age of mice.

The normal values of BUN and creatinine obtained from the 14 treated asymptomatic animals, compared with the 2 infected control, also support the histological findings. These results and the high serum antitoxin levels reflect a high degree of survival and robust protection against long-term tissue damage. Unfortunately, the small number of surviving infected control mice was insufficient to measure renal function, as most of them died overnight.

### Histological Examination

**Figure 4.**
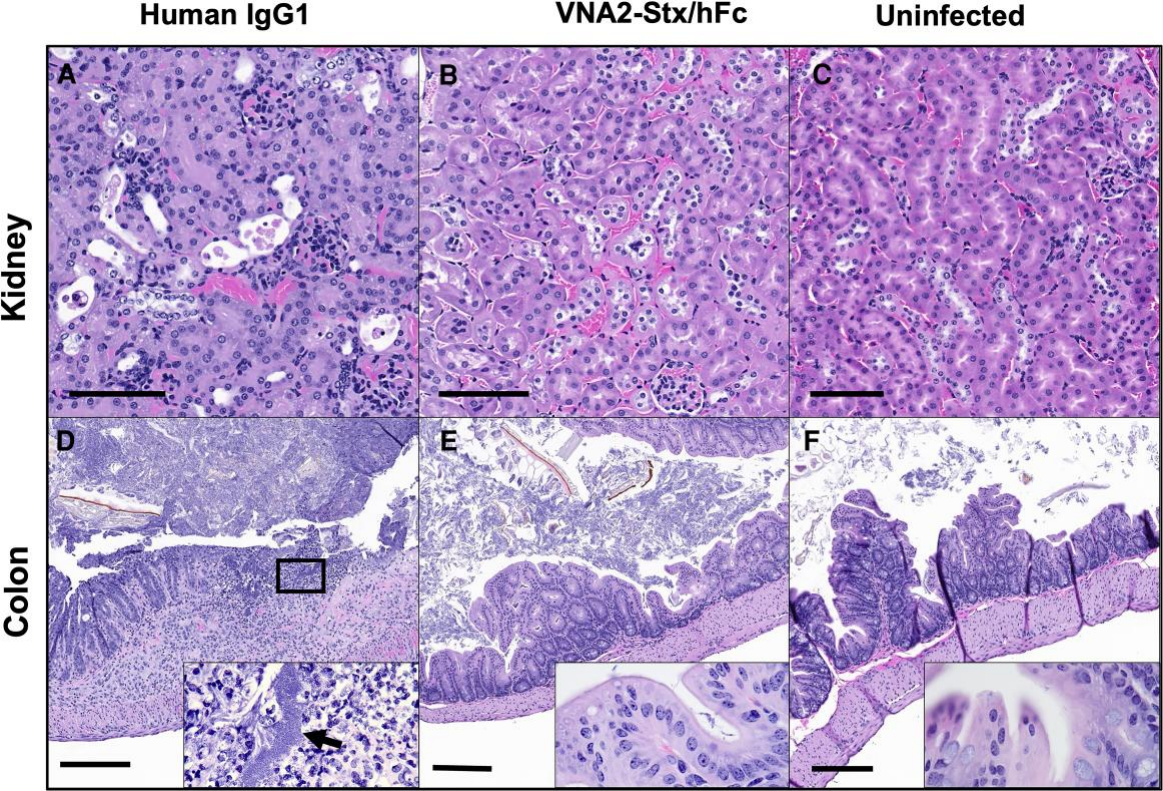
VNA treatment reduces renal and colonic pathology associated with *Citrobacter rodentium* (*Cr*) infection in mice. Mice orally infected with *Cr-Stx2d* were treated 2 days PI with either a human IgG1 isotype control (*A* and *D*) and euthanized on day 8 PI or with VNA2-Stx/hFc (*B* and *E*), all to be compared with uninfected controls (*C* and *F*) that were euthanized on day 21 PI. In representative mice, pathology in the kidney (*A*) was characterized by dilation of proximal renal tubules with sloughing of renal tubular epithelium into tubule lumina (casts), whereas tubules from VNA2-Stx/hFc-treated animals showed only mild vacuolation of the proximal tubule epithelium (*B*) that was indistinguishable from kidneys from uninfected animals (*C*). Regions of severe ulceration and expansion of the lamina propria and submucosa by macrophages and lymphocytes, with adhered bacterial colonies (black box, arrow), was noted in the isotype control treated animal (*D*), whereas the colon from the VNA2-Stx/hFc (*E*) and uninfected (*F*) animals showed no evidence of colonic pathology with normal colonic epithelium. Hematoxylin and eosin; scale bars = 100 μM (*A–C*); 200 μM, inset 100× oil (*D–F*). Abbreviations: hFc, human IgG1 Fc domain;; PI, postinfection; Stx, Shiga toxin; VNA2, Variable Heavy domain of a Heavy-chain only antibody neutralizing agent 2.

Figure 4 clearly demonstrates that VNA2-Stx/hFc treatment prevented renal damage and reduced colonic pathology in *Cr*-Stx2d–infected mice. Figure 4*A*–*C* shows sections from the kidneys and Figure 4*D*–*F* shows sections from the colons of mice in each of the following groups: (1) the infected control group (Figure 4*A* and 4*D*), (2) the group treated with VNA2-Stx/hFc 2 days following *Cr*-Stx2d infection (Figure 4*B* and 4*E*), and (3) the uninfected control group (Figure 4*C* and 4*F*). The infected control mice reached terminal endpoint at 8 days following infection. Pathology in the kidney (Figure 4*A*) was characterized by dilation of proximal renal tubules with sloughing of renal tubular epithelium into tubule lumina (casts), whereas tubules from VNA2-Stx/hFc-treated animals (21 days postinfection) showed only mild vacuolation of the proximal tubule epithelium (Figure 4*B*). The results shown in Figure 4*B* were virtually indistinguishable from those of the kidneys of the uninfected animals (Figure 4*C*). Regions of severe ulceration and expansion of the lamina propria and submucosa by macrophages and lymphocytes were noted in the control IgG1-treated animal (Figure 4*D*), whereas the colons from the VNA2-Stx/hFc-treated (Figure 4*E*) and uninfected (Figure 4*F*) animals show no evidence of colonic pathology with normal colonic epithelium.

## DISCUSSION

Since antibiotics are contraindicated for STEC infections [6], this pathogen remains a serious medical risk to patients. Attempts to develop antitoxin countermeasures against HUS include human monoclonal antibodies (HmAbs) [21] and the humanized monoclonal antibody TMA-15 [6], which was evaluated in phase 1 clinical trials. The HmAb targeting Stx1 and Stx2a was shown to be highly protective in different animal STEC disease models including piglets treated 24–48 hours post–oral STEC challenge [7, 8, 11, 13]. However, due to serious logistical obstacles, these products were not pursued further. We consequently developed an Stx-neutralizing heterotrimeric antitoxin, VNA1-Stx, consisting of 3 linked VHHs, which was highly protective in the mouse toxicity model with Stx1 and/or Stx2a when coadministered with an effector antibody to promote toxin clearance [20].

Here we describe the development of an improved VNA agent, VNA2-Stx/hFc, in which the combination of 4 different VHHs provides a broader natural Stx variant specificity and at least equivalent potency to VNA1-Stx [20], and contains a human Fc domain (hFc) for longer-term serum persistence. More recent studies demonstrate that the VHH components of VNA1-Stx displayed poor binding to the distantly related, and medically important, Stx2d variant (which has a B subunit identical to Stx2c). We thus rescreened our VHH display phage library and identified new Stx-neutralizing VHHs showing broad specificity for the B subunits of both Stx2a and Stx2d. We thus added 2 of the new VHHs to VNA1-Stx heterotrimer, replacing one, to create a new VHH heterotetramer, VNA2-Stx. Finally, we showed that VNA2-Stx potently neutralized Stx1, Stx2a, and Stx2d natural variants and was highly protective in the mouse toxicity model when administered formulated as replicon RNA [23].

These observations prompted us to examine the therapeutic efficacy of VNA2-Stx/hFc in the *Cr*-Stx2d oral infection mouse model [17–20], which induces bacterial A/E lesions in the gut mucosa, systemic uptake of Stx2d, and fatal kidney damage to the proximal renal tubules. The model is thus similar to STEC in humans where the A/E lesions and systemic uptake of Stx lead to damage to the kidney glomeruli and sometimes HUS. Because of the use of 4 different VNA2-Stx/hFc components, each binding to different epitopes and each displaying varying levels of cross-variant specificity, this molecule is likely to have at least some potency for virtually all STEC natural variants of concern to humans.

The prodromal interval of 3–7 days between the onset of diarrhea and STEC-HUS in patients offers a short window of opportunity for disease intervention and protection to contacts and other individuals exposed to the source of infection [6, 22]. Similarly, the *Cr*-Stx2d model has a long course of infection in which mice display distinct pathology measured as weight loss about 4–7 days postinfection. As such, this model may be more relevant to human STEC infections than the earlier described mouse toxicity model [23]. The results reported here show that a single treatment of VNA2-Stx/hFc protected all mice when administered 4 days, and sometimes even 5 days, after *Cr*-Stx2d challenge.

At euthanasia, all treated mice had appreciable levels of serum VHH antitoxin, more than 2 weeks posttreatment, and all infected mice showed extensive bacterial excretion of *Cr*-Stx2d in the feces, regardless of treatment. Histological sections demonstrate extensive gut mucosal lesions, and serious kidney lesions, in contrast to mice treated with control IgG1 (Figure 4). The normal values of BUN and creatinine obtained from the 14 treated asymptomatic animals, compared with the 2 infected control animals, also supported the histological findings. These results and the 100% survival reflect the robust protection against long-term tissue damage. Together, our data suggest that a potent Stx antitoxin, such as VNA2-Stx/hFc, provided to STEC patients near the time of symptom onset may prevent some or all of the serious systemic consequences of this disease.

It is unclear why females displayed a longer treatment window of up to 5 days postinfection compared to 4 days for males. Nevertheless, our results strongly suggest that treatment of patients at the onset of bloody diarrhea may prevent or minimize HUS sequelae in humans. The treatment will likely provide prophylactic protection to contact individuals and to asymptomatic people exposed to a source of infection.

In summary, using a genetically modified *Cr* that infects the gut mucosa of mice causing mucosal damage, and the systemic uptake of liberated Stx causing fatal renal damage, we demonstrate the highly specific efficacy of VNA2-Stx/hFc in the protection of mice against fatal disease. In light of the current lack of specific therapeutic agents to treat or prevent STEC-HUS in humans, a broad-specificity, Stx antitoxin agent such as VNA2-Stx/hFc potentially offers a credible remedy.

## References

[1] Milford DV, Taylor CM, Guttridge B, et al Haemolytic uraemic syndromes in the British Isles 1985-8: association with verocytotoxin producing *Escherichia coli*. Part 1: Clinical and epidemiological aspects. Arch Dis Child 1990; 65:716–21. 2201261 10.1136/adc.65.7.716 PMC1792437

[2] Boerlin P, McEwen SA, Boerlin-Petzold F, et al Associations between virulence factors of Shiga toxin–producing *Escherichia coli* and disease in humans. J Clin Microbiol 1999; 37:497–503. 9986802 10.1128/jcm.37.3.497-503.1999 PMC84443

[3] Ostroff SM, Tarr PI, Neill MA, et al Toxin genotypes and plasmid profiles as determinants of systemic sequelae in *Escherichia coli* O157:H7 infections. J Infect Dis 1989; 160:994–8. 2685131 10.1093/infdis/160.6.994

[4] Griffin PM, Tauxe RV. The epidemiology of infections caused by *Escherichia coli* O157:H7, other enterohemorrhagic *E. coli*, and the associated hemolytic uremic syndrome. Epidemiol Rev 1991; 13:60–98. 1765120 10.1093/oxfordjournals.epirev.a036079

[5] Wang X, Yu D, Chui L, et al A comprehensive review on Shiga toxin subtypes and their niche-related distribution characteristics in Shiga-toxin–producing *E. coli* and other bacterial hosts. Microorganisms 2024; 12:687. 38674631 10.3390/microorganisms12040687 PMC11052178

[6] Tzipori S, Sheoran A, Akiyoshi D, et al Antibody therapy in the management of shiga toxin-induced hemolytic uremic syndrome. Clin Microbiol Rev 2004; 17:926–41. 15489355 10.1128/CMR.17.4.926-941.2004 PMC523565

[7] Mukherjee J, Chios K, Fishwild D, et al Human Stx2-specific monoclonal antibodies prevent systemic complications of *Escherichia coli* O157:H7 infection. Infect Immun 2002; 70:612–9. 11796590 10.1128/iai.70.2.612-619.2002 PMC127659

[8] Mukherjee J, Chios K, Fishwild D, et al Production and characterization of protective human antibodies against Shiga toxin 1. Infect Immun 2002; 70:5896–9. 12228326 10.1128/IAI.70.10.5896-5899.2002 PMC128343

[9] Sheoran AS, Chapman S, Singh P, et al Stx2-specific human monoclonal antibodies protect mice against lethal infection with *Escherichia coli* expressing Stx2 variants. Infect Immun 2003; 71:3125–30. 12761090 10.1128/IAI.71.6.3125-3130.2003 PMC155773

[10] Sauter KAD, Melton-Celsa AR, Larkin K, et al Mouse model of hemolytic-uremic syndrome caused by endotoxin-free Shiga toxin 2 (Stx2) and protection from lethal outcome by anti-Stx2 antibody. Infect Immun 2008; 76:4469–78. 18694970 10.1128/IAI.00592-08 PMC2546846

[11] Wadolkowski EA, Sung LM, Burris JA, et al Acute renal tubular necrosis and death of mice orally infected with *Escherichia coli* strains that produce Shiga-like toxin type II. Infect Immun 1990; 58:3959–65. 2254023 10.1128/iai.58.12.3959-3965.1990 PMC313762

[12] Sheoran AS, Chapman-Bonofiglio S, Harvey BR, et al Human antibody against shiga toxin 2 administered to piglets after the onset of diarrhea due to *Escherichia coli* O157:H7 prevents fatal systemic complications. Infect Immun 2005; 73:4607–13. 16040972 10.1128/IAI.73.8.4607-4613.2005 PMC1201267

[13] Jeong K-I, Tzipori S, Sheoran AS. Shiga toxin 2–specific but not Shiga toxin 1–specific human monoclonal antibody protects piglets challenged with enterohemorrhagic *Escherichia coli* producing Shiga toxin 1 and Shiga toxin 2. J Infect Dis 2010; 201:1081–3. 20196656 10.1086/651198 PMC2846416

[14] Sheoran AS, Dmitriev IP, Kashentseva EA, et al Adenovirus vector expressing Stx1/Stx2-neutralizing agent protects piglets infected with *Escherichia coli* O157:H7 against fatal systemic intoxication. Infect Immun 2015; 83:286–91. 25368111 10.1128/IAI.02360-14 PMC4288880

[15] Saif LJ, Ward LA, Yuan L, et al The gnotobiotic piglet as a model for studies of disease pathogenesis and immunity to human rotaviruses. Arch Virol Suppl 1996; 12:153–61. 9015112 10.1007/978-3-7091-6553-9_17

[16] Steele J, Feng H, Parry N, et al Piglet models of acute or chronic *Clostridium difficile* illness. J Infect Dis 2010; 201:428–34. 20039803 10.1086/649799 PMC2804769

[17] Zhang Q, Widmer G, Tzipori S. A pig model of the human gastrointestinal tract. Gut Microbes 2013; 4:1–8. 10.4161/gmic.23867 PMC3669164 23549377

[18] Mallick EM, McBee ME, Vanguri VK, et al A novel murine infection model for Shiga toxin–producing *Escherichia coli*. J Clin Invest 2012; 122:4012–24. 23041631 10.1172/JCI62746 PMC3484439

[19] Flowers LJ, Hu S, Shrestha A, et al *Citrobacter rodentium* lysogenized with a Shiga toxin-producing phage: a murine model for Shiga toxin–producing *E. coli* infection. Methods Mol Biol 2021; 2291:381–97. 33704765 10.1007/978-1-0716-1339-9_19

[20] Bowser S, Melton-Celsa A, Chapartegui-González I, et al Further evaluation of enterohemorrhagic *Escherichia coli* gold nanoparticle vaccines utilizing *Citrobacter rodentium* as the model organism. Vaccines (Basel) 2024; 12:508. 38793759 10.3390/vaccines12050508 PMC11125983

[21] Tremblay JM, Mukherjee J, Leysath CE, et al A single VHH-based toxin-neutralizing agent and an effector antibody protect mice against challenge with Shiga toxins 1 and 2. Infect Immun 2013; 81:4592–603. 24082082 10.1128/IAI.01033-13 PMC3837998

[22] Robinson SR, Dayao DA, Medina JA, et al An anti-Shiga toxin VHH nanobody multimer protects mice against fatal toxicosis when administered intramuscularly as repRNA. Infect Immun 2024; 92:e0023924. 39392311 10.1128/iai.00239-24 PMC11556087

[23] Paton AW, Morona R, Paton JC. A new biological agent for treatment of Shiga toxigenic *Escherichia coli* infections and dysentery in humans. Nat Med 2000; 6:265–70. 10700227 10.1038/73111

